# A Genetic and Pharmacological Analysis of Isoprenoid Pathway by LC-MS/MS in Fission Yeast

**DOI:** 10.1371/journal.pone.0049004

**Published:** 2012-11-07

**Authors:** Tomonori Takami, Yue Fang, Xin Zhou, Wurentuya Jaiseng, Yan Ma, Takayoshi Kuno

**Affiliations:** 1 Division of Molecular Pharmacology and Pharmacogenomics, Department of Biochemistry and Molecular Biology, Kobe University Graduate School of Medicine, Kobe, Japan; 2 Chemical Analysis Section, JCL Bioassay Corporation, Nishiwaki, Japan; 3 Department of Pharmacology, School of Pharmaceutical Sciences, China Medical University, Shenyang, China; 4 The First Affiliated Hospital of Liaoning Medical University, Jinzhou, China; Cancer Research UK London Research Institute, United Kingdom

## Abstract

Currently, statins are the only drugs acting on the mammalian isoprenoid pathway. The mammalian genes in this pathway are not easily amenable to genetic manipulation. Thus, it is difficult to study the effects of the inhibition of various enzymes on the intermediate and final products in the isoprenoid pathway. In fission yeast, antifungal compounds such as azoles and terbinafine are available as inhibitors of the pathway in addition to statins, and various isoprenoid pathway mutants are also available. Here in these mutants, treated with statins or antifungals, we quantified the final and intermediate products of the fission yeast isoprenoid pathway using liquid chromatography-mass spectrometry/mass spectrometry. In *hmg1-1*, a mutant of the gene encoding 3-hydroxy-3-methylglutaryl coenzyme A reductase (HMGR), ergosterol (a final sterol product), and squalene (an intermediate pathway product), were decreased to approximately 80% and 10%, respectively, compared with that of wild-type cells. Consistently in wild-type cells, pravastatin, an HMGR inhibitor decreased ergosterol and squalene, and the effect was more pronounced on squalene. In *hmg1-1* mutant and in wild-type cells treated with pravastatin, the decrease in the levels of farnesyl pyrophosphate and geranylgeranyl pyrophosphate respectively was larger than that of ergosterol but was smaller than that of squalene. In Δ*erg6* or Δ*sts1* cells, mutants of the genes involved in the last step of the pathway, ergosterol was not detected, and the changes of intermediate product levels were distinct from that of *hmg1-1* mutant. Notably, in wild-type cells miconazole and terbinafine only slightly decreased ergosterol level. Altogether, these studies suggest that the pleiotropic phenotypes caused by the *hmg1-1* mutation and pravastatin might be due to decreased levels of isoprenoid pyrophosphates or other isoprenoid pathway intermediate products rather than due to a decreased ergosterol level.

## Introduction

The isoprenoid pathway is essential for all organisms. Regulation of the isoprenoid pathway has been extensively studied in mammals for many years, because this pathway produces such critical end-products as steroid hormones, cholesterol and bile acids [1]. In eukaryotes, the biosynthesis of isoprenoids occurs through the mevalonate pathway which starts with the biosynthesis of acetoacetyl coenzyme A and the subsequent reactions lead to the biosynthesis of mevalonate. In the following steps, mevalonate is transformed into farnesyl pyrophosphate (FPP), a branch-point of the pathway that serves as a substrate for enzymes that synthesize sterol and nonsterol products (i.e. dolichols, ubiquinones and heme A) as well as prenyl groups for post-translational modification of proteins [2]. Ubiquinone involves electron transfer system that affects energy metabolisms [3] and dolichol involves glycosylation of proteins [2].

Statins are selective inhibitors of 3-hydroxy-3-methylglutaryl coenzyme A reductase (HMGR), which inhibit the biosynthesis of cholesterol and thereby reduce serum cholesterol levels in humans. In addition to the inhibition of cholesterol synthesis, statins have been shown to possess anti-inflammatory and immune-modulatory pleiotropic effects, even in patients with normal cholesterol levels [4]. The immediate product of HMGR is mevalonate, which is metabolized into the nonsterol isoprenoids FPP and geranylgeranyl pyrophosphate (GGPP), and cholesterol in mammals. FPP and GGPP are necessary for the post-translational isoprenylation of monomeric small GTP-binding proteins that are involved in many important biological processes. Statins attenuate synthesis of not only cholesterol but also isoprenoid pyrophosphates. Thus, the pleiotropic effects of statins are thought to be mediated partly via inhibition of isoprenoid pyrophosphates synthesis [5].

In unicellular eukaryotes such as *Schizosaccharomyces pombe* (*S. pombe*), the primary role of the isoprenoid pathway is the synthesis of the essential sterol and nonsterol isoprenoids. Nonsterol isoprenoids are same as that of mammals, but the essential sterol is ergosterol, the main sterol of most higher fungi [6]. Some of the antifungal drugs are known to inhibit ergosterol biosynthesis pathway enzymes. Terbinafine, an allylamine, inhibits the *erg1* ^+^ product squalene epoxidase [7], which acts upstream of the *erg11* ^+^ product lanosterol demethylase that is inhibited by azoles [8]. Therefore, fission yeast (*S. pombe*) provides a useful model system to study the regulation mechanism of isoprenoid pathway. We previously isolated an allele of the essential *hmg1* ^+^ gene encoding HMGR, *hmg1-1*, as a mutant that showed hypersensitivities to high temperature and to FK506, a calcineurin inhibitor [9]. The *hmg1-1* allele contained an opal nonsense mutation in its N-terminal transmembrane domain, yet in spite of the mutation a full-length protein was produced. We also showed that the amount of the mutated gene tagged with GFP protein was lower (approximately 30–50%) than the wild-type protein expressed in wild-type cells by immunoblot analysis [9]. The *hmg1-1* mutant showed hypersensitivity to pravastatin, an HMGR inhibitor, suggesting it has defective HMGR activity. In particular, the mutant showed defects in cell wall integrity and exhibited different phenotypes from those of the disruption mutants of ergosterol biosynthesis genes, and it showed normal filipin staining as well as normal subcellular localization of small GTPases. These data suggest that the pleiotropic phenotypes reflect the integrated effects of the reduced availability of ergosterol as well as various intermediates of the isoprenoid pathway [9].

Here, we quantified the final product (ergosterol) and the pathway intermediates (squalene, FPP, GGPP, and lanosterol) in various isoprenoid pathway mutants, treated with statins or antifungals using liquid chromatography-mass spectrometry/mass spectrometry (LC-MS/MS). The results showed that compounds such as pravastatin, allylamine terbinafine, and miconazole inhibit Hmg1, squalene epoxidase (Erg1), and lanosterol demethylase (Erg11), respectively, and the inhibition was associated with significant changes in the levels of the pathway products and intermediates. Notably, the ergosterol level showed substantial changes but the changes were smaller in magnitude when compared with FPP and GGPP in response to these drugs.

## Results

### Validity of measurement

Selected reaction monitoring (SRM) chromatograms of squalene, lanosterol, ergosterol, pyrene (used as an internal standard), FPP, and GGPP in the standard solution are shown in Figure S1. These conditions gave sharp peaks for each compound and showed a good separation of each peak. The calibration curves of squalene, lanosterol, FPP, and GGPP in the standard solution are shown in Figure S2. The calibration curves of squalene, lanosterol, FPP, and GGPP were constructed in the range of 1–500 µmol/l, 0.1–10 µmol/l, 10–400 nmol/l, and 10–400 nmol/l, respectively. The calibration curves of all the compounds displayed correlation coefficients (*r*) higher than 0.990.

The SRM chromatograms of ergosterol and pyrene in the blank sample, the zero sample, and the lower limit of quantification (LLOQ) sample are shown in Figure S3. The conditions gave sharp peaks for ergosterol and pyrene and showed a good separation from the endogenous peaks. The calibration curve of ergosterol was constructed in the range of 1.9–380 nmol/mg protein. The correlation coefficient of the calibration curve of ergosterol is 0.9967 (Figure S4). These results suggest that the LC-MS/MS system used in this study enabled absolute quantification of ergosterol and relative quantification of squalene, lanosterol, FPP, and GGPP.

### Levels of ergosterol, squalene, FPP, and GGPP in Δ*sts1* and Δ*spo9* cells

The *sts1* ^+^ gene encodes C-24 (28) sterol reductase that catalyzes the final step in ergosterol biosynthesis [6]. Consistently, no ergosterol peak was observed in Δ*sts1* cells. Therefore absolute ergosterol was quantified by constructing a calibration curve using chloroform/methanol (2∶1, v/v) extract from Δ*sts1* cells as a blank sample. The concentration of ergosterol in wild-type cells was determined to be 23.0±2.6 nmol/mg protein (n = 5 experiments).

**Figure 1:**
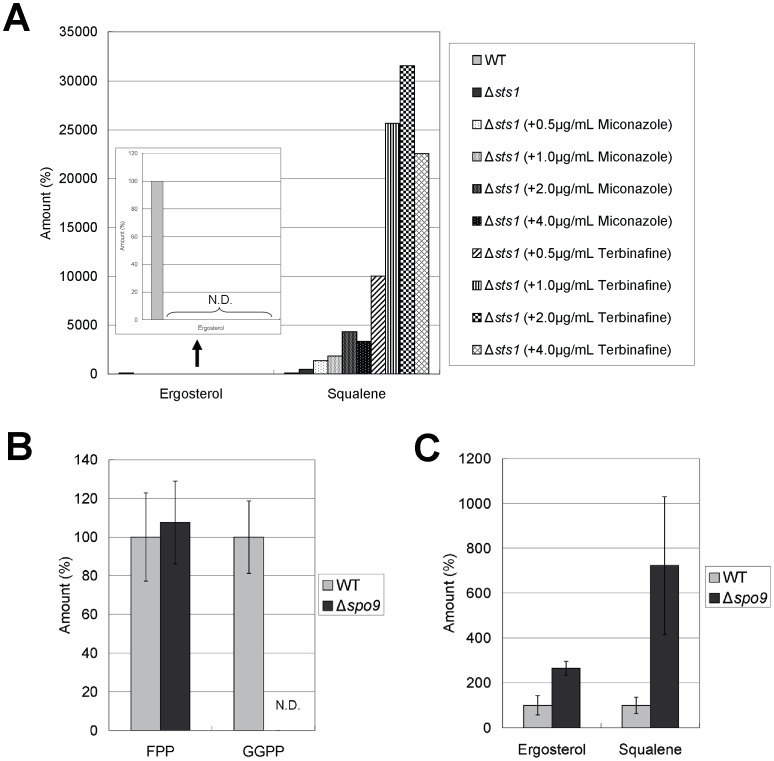
Levels of squalene, ergosterol, FPP, and GGPP. (A) Levels of squalene and ergosterol in wild-type cells and Δ*sts1* cells treated with terbinafine or miconazole. Wild-type and Δ*sts1* cells were grown to saturation at 27°C in liquid YPD medium. Cells were further incubated at 27°C for 10 h in the absence or presence of terbinafine (0.5, 1.0, 2.0, 4.0 µg/ml) or miconazole (0.5, 1.0, 2.0, 4.0 µg/ml) as indicated, and then squalene and ergosterol of the strains were extracted and determined as described in Materials and Methods. The data shown are representative of multiple experiments. N.D. represents “not detected”. (B) Levels of FPP and GGPP in wild-type cells and Δ*spo9* cells. Wild-type and Δ*spo9* cells were grown to saturation at 27°C in liquid YPD medium, and then FPP and GGPP of the strains were extracted and determined. Error bars represent standard deviations (n = 3 experiments). N.D. represents “not detected”. (C) Levels of squalene and ergosterol in wild-type cells and Δ*spo9* cells. Wild-type and Δ*spo9* cells were grown to saturation at 27°C in liquid YPD medium, and then squalene and ergosterol of the strains were extracted and determined. Error bars represent standard deviations (n = 3 experiments).

**Figure 2:**
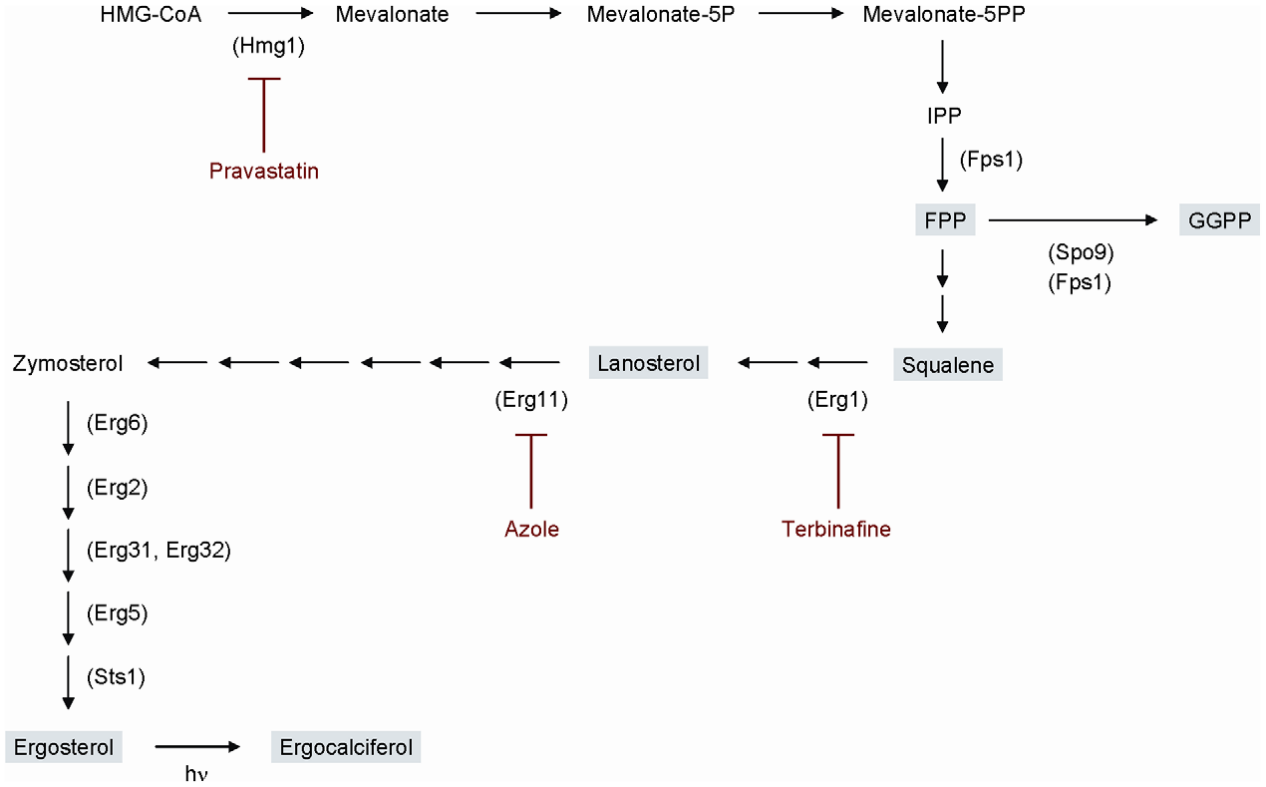
Isoprenoid pathway in *S. pombe.* Enzymes predicted to be involved in ergosterol biosynthesis are shown in parentheses with key intermediates in gray boxes. Inhibitors are shown by red letters. The *S. pombe* ergosterol synthesis pathway is deduced from that of *S. cerevisiae* [6], [10].

The quantification results of squalene and ergosterol in wild-type cells and Δ*sts1* cells treated with terbinafine or miconazole are shown in Figure 1A. Four- to five-fold-higher squalene levels were detected in Δ*sts1* cells compared with that in wild-type cells. Furthermore, the squalene levels in Δ*sts1* drastically increased up to 40-fold and 300-fold by addition of miconazole and terbinafine to the medium, respectively. These results are in good agreement with the fission yeast isoprenoid pathway shown in Figure 2 suggested by other researchers [6], [10].

The *spo9* ^+^ gene encodes geranylgeranyl pyrophosphate synthetase that is a key enzyme in isoprenoid biosynthesis [11]. We then examined the effect of *spo9* deletion on the levels of the intermediates (squalene, FPP, and GGPP) and the final sterol product (ergosterol) in the isoprenoid pathway. Results showed that FPP level in Δ*spo9* cells was almost equal to that in wild-type cells, however, GGPP was not detected in Δ*spo9* cells (Figure 1B). Consistent with these results, Ye *et al*. showed that Fps1 acts mainly as FPP synthase and that the heteromer of Spo9 and Fps1 acts as GGPP synthase in fission yeast [11]. On the other hand, Figure 1C showed that levels of ergosterol and squalene in Δ*spo9* cells were 264% and 722%, respectively, compared with that of wild-type cells.

### Levels of squalene, ergosterol, FPP, and GGPP in *hmg1-1* mutant and wild-type cells treated with pravastatin

**Figure 3:**
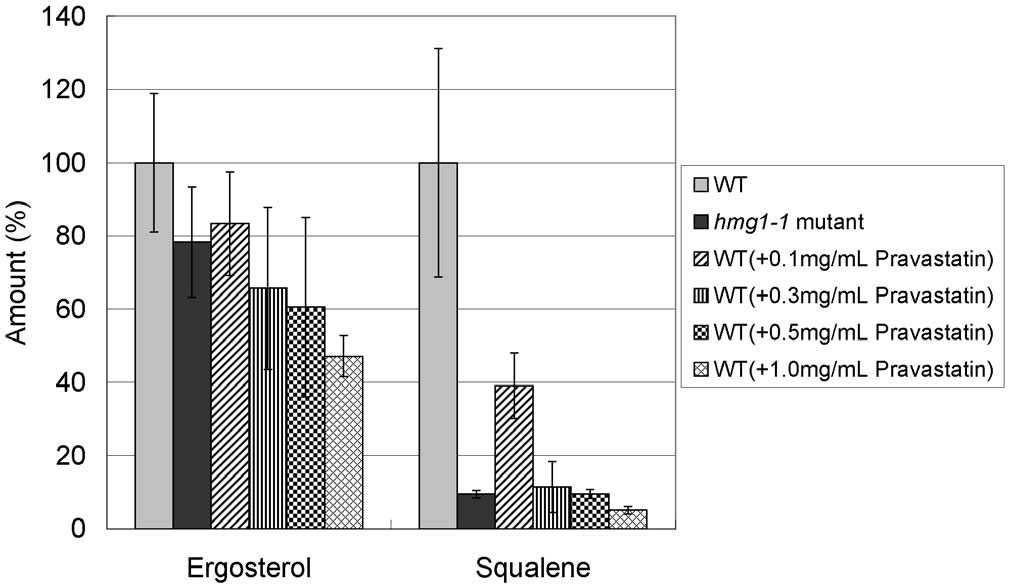
Changes in the levels of squalene and ergosterol in *hmg1-1* mutant and wild-type cells treated with pravastatin. Wild-type and *hmg1-1* mutants were grown to saturation at 27°C in liquid YPD medium. Cells were further incubated at 27°C for 10 h in the absence or presence of pravastatin (0.1, 0.3, 0.5, 1.0 mg/ml) and then squalene and ergosterol of the strains were extracted and determined. Error bars represent standard deviations (n = 3 experiments).

Next, we measured levels of squalene and ergosterol in *hmg1-1* mutant and wild-type cells treated with pravastatin. Strikingly, results showed that squalene level in *hmg1-1* mutant was decreased to only 10% of that of wild-type cells, whereas ergosterol level in *hmg1-1* mutant was decreased to 78% of that of wild-type cells (Figure 3). These tendencies were the same for wild-type cells treated with pravastatin (Figure 3).

**Figure 4:**
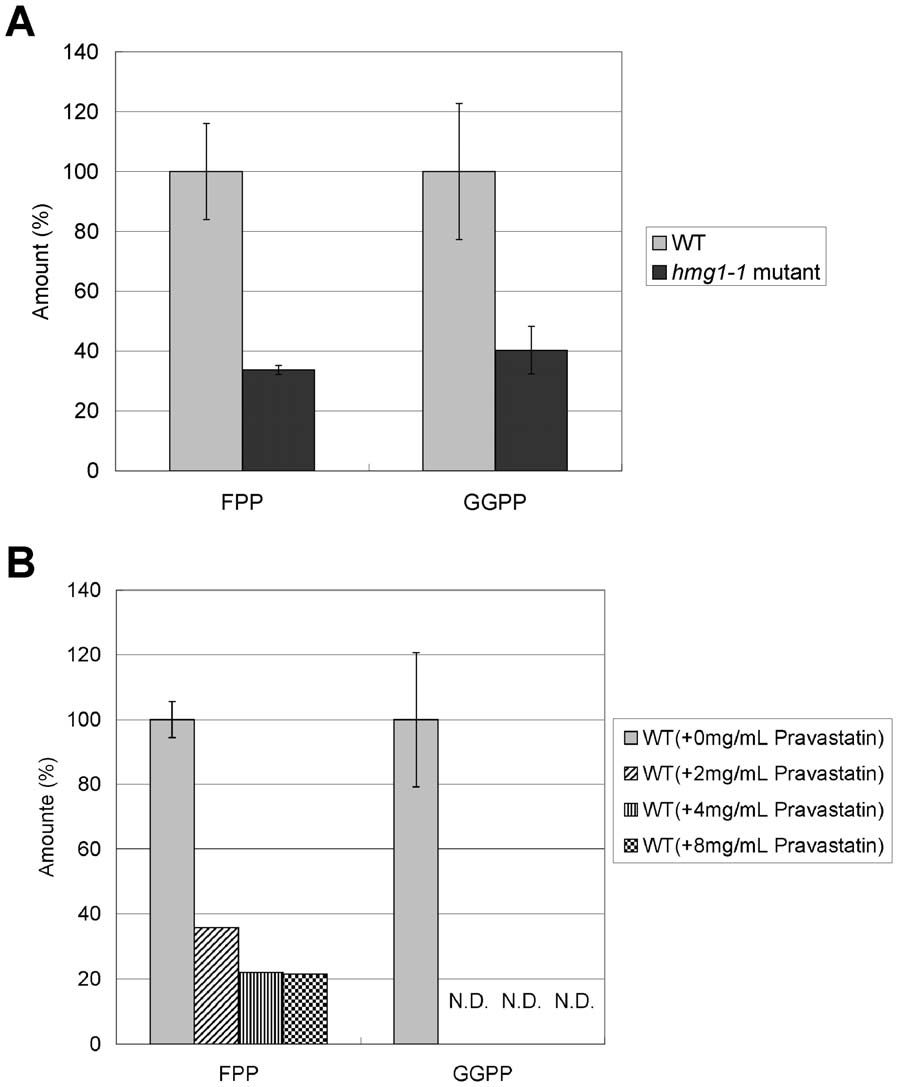
Levels of FPP and GGPP. (A) Levels of FPP and GGPP in wild-type cells and *hmg1-1* mutant. Wild-type cells and *hmg1-1* mutants were grown to saturation at 27°C in liquid YPD medium, and then FPP and GGPP of the strains were extracted and determined. Error bars represent standard deviations (n = 3 experiments). (B) Levels of FPP and GGPP in wild-type cells treated with pravastatin. Wild-type cells were grown to saturation at 27°C in liquid YPD medium. Cells were further incubated at 27°C for 10 h in the absence or presence of pravastatin (2.0, 4.0, 8.0 mg/ml) and then FPP and GGPP of the strains were extracted and determined. Error bars represent standard deviations (n = 3 experiments). N.D. represents “not detected”.

We also measured levels of FPP and GGPP in *hmg1-1* mutant and wild-type cells treated with pravastatin. As shown in Figure 4A, levels of FPP and GGPP in *hmg1-1* mutant were 34% and 40% of that of wild-type cells, respectively (Figure 4A). Moreover, FPP levels in wild-type cells treated with high concentration of pravastatin (>2 mg/ml) were 20%–40% of that seen in untreated wild-type cells similar to that in *hmg1-1* mutant, whereas GGPP was not detected in wild-type cells treated with pravastatin (Figure 4B).

### Effects of terbinafine and miconazole on the levels of squalene, lanosterol, and ergosterol in various isoprenoid pathway mutants

**Figure 5:**
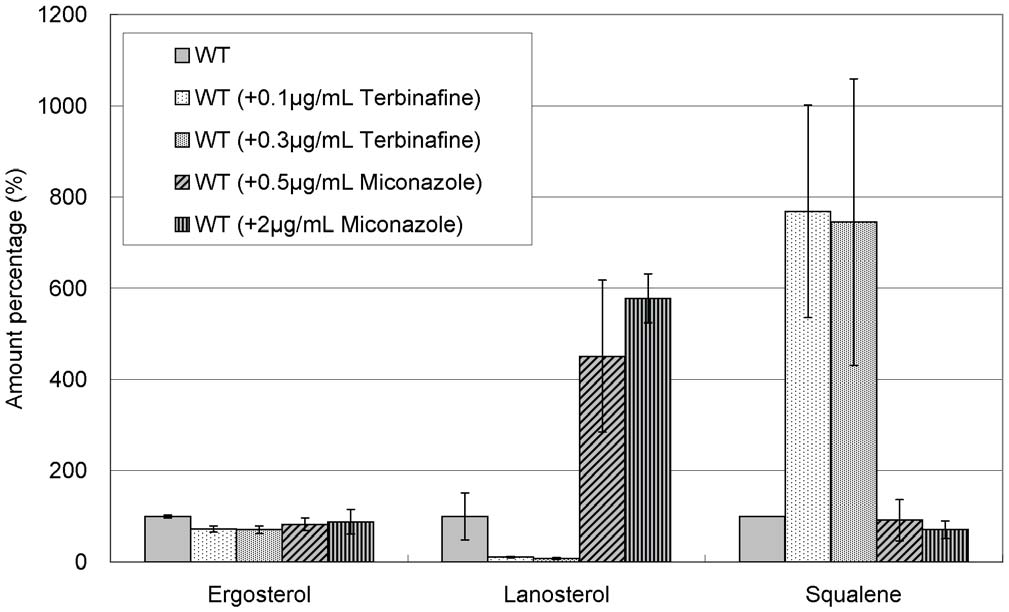
Effect of terbinafine and miconazole treatment on levels of ergosterol, lanosterol, and squalene in wild-type cells. Wild-type cells were grown to saturation at 27°C in liquid YPD medium. Cells were further incubated at 27°C for 10 h in the absence or presence of terbinafine (0.1, 0.3 µg/ml) or miconazole (0.5, 2.0 µg/ml) as indicated, and then ergosterol, lanosterol, and squalene of the strains were extracted and determined. Error bars represent standard deviations (n = 3 experiments).

We first examined the effects of terbinafine and miconazole in wild-type cells. As expected, terbinafine significantly decreased lanosterol level and increased squalene level in wild-type cells (Figure 5). In contrast, miconazole dramatically increased lanosterol level but only slightly affected squalene level in wild-type cells. Notably, ergosterol levels in wild-type cells were only slightly affected by both drugs (Figure 5).

Then, we examined the effects of these drugs on the levels of ergosterol, lanosterol, and squalene in various isoprenoid pathway mutants including *hmg1-1* mutant, Δ*erg6*, Δ*erg31*Δ*erg32*, and Δ*erg5* cells, respectively. The *erg6* ^+^ gene encodes sterol 24-C-methyltransferase Erg6; the *erg31* ^+^ gene and the *erg32* ^+^ gene encode C-5 sterol desaturase; the *erg5* ^+^ gene encodes C-22 sterol desaturase Erg5 [8]. All of them were responsible for catalyzing a sequence of reactions from zymosterol to ergosterol.

**Figure 6:**
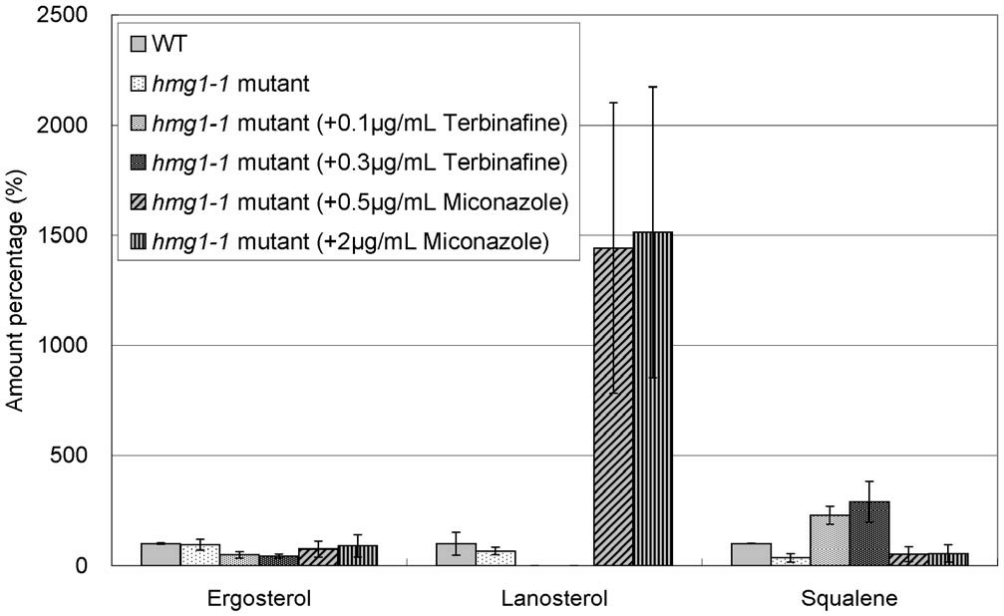
Effect of terbinafine and miconazole treatment on levels of ergosterol, lanosterol, and squalene in *hmg1-1* mutant. Wild-type cells and *hmg1-1* mutants were grown to saturation at 27°C in liquid YPD medium. Cells were further incubated at 27°C for 10 h in the absence or presence of terbinafine (0.1, 0.3 µg/ml) or miconazole (0.5, 2.0 µg/ml) as indicated, and then ergosterol, lanosterol, and squalene of the strains were extracted and determined. Error bars represent standard deviations (n = 3 experiments).

**Figure 7:**
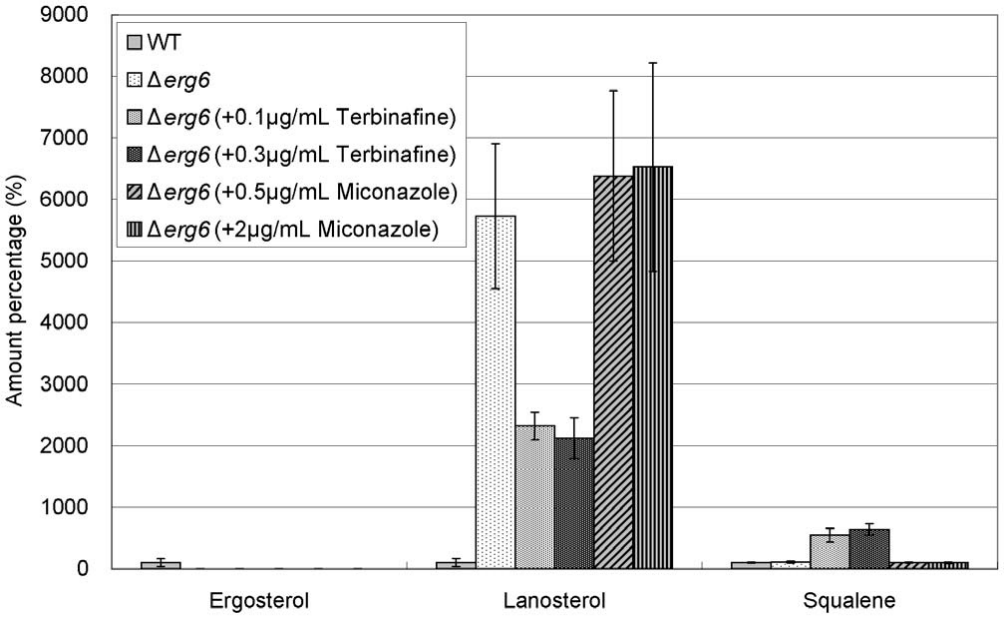
Effect of terbinafine and miconazole treatment on levels of ergosterol, lanosterol, and squalene in Δ*erg6* cells. Wild-type cells and Δ*erg6* cells were grown to saturation at 27°C in liquid YPD medium. Cells were further incubated at 27°C for 10 h in the absence or presence of terbinafine (0.1, 0.3 µg/ml) or miconazole (0.5, 2.0 µg/ml) as indicated, and then ergosterol, lanosterol, and squalene of the strains were extracted and determined. Error bars represent standard deviations (n = 3 experiments).

**Figure 8:**
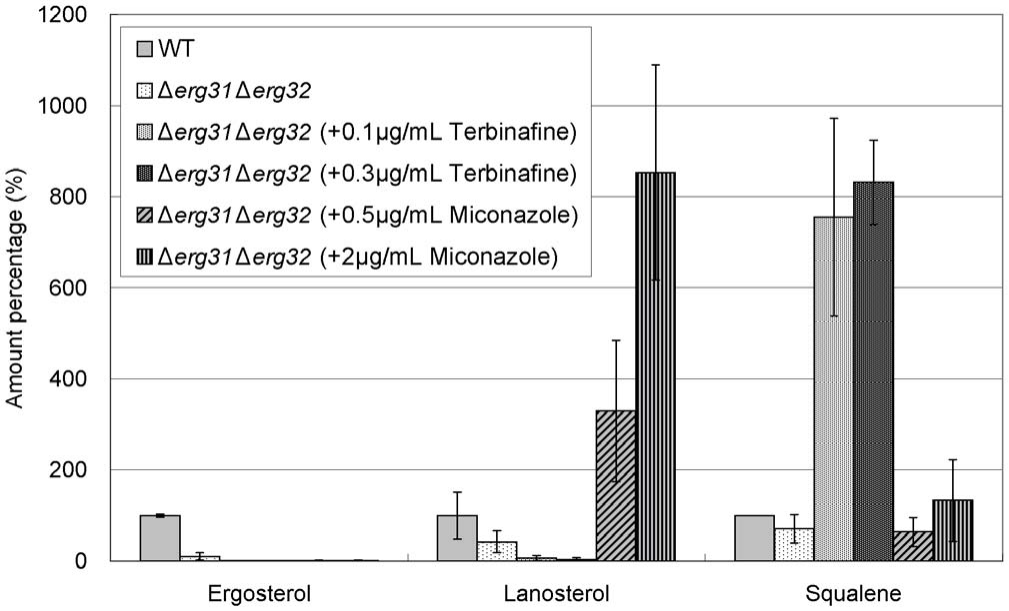
Effect of terbinafine and miconazole treatment on levels of ergosterol, lanosterol, and squalene in Δ*erg31*Δ*erg32* cells. Wild-type cells and Δ*erg31*Δ*erg32* cells were grown to saturation at 27°C in liquid YPD medium. Cells were further incubated at 27°C for 10 h in the absence or presence of terbinafine (0.1, 0.3 µg/ml) or miconazole (0.5, 2.0 µg/ml) as indicated, and then ergosterol, lanosterol, and squalene of the strains were extracted and determined. Error bars represent standard deviations (n = 3 experiments).

**Figure 9:**
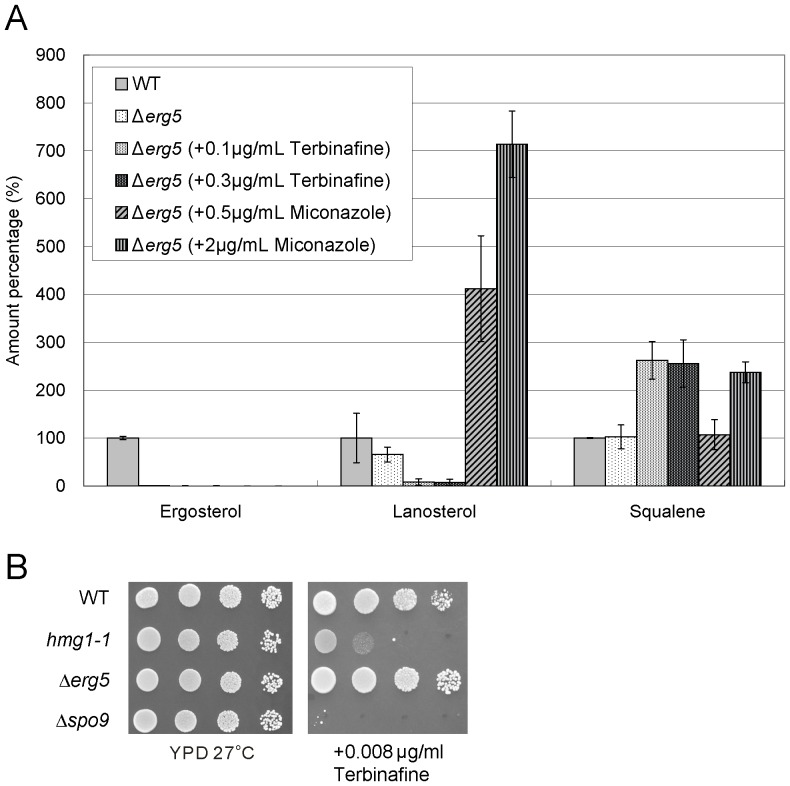
Effect of terbinafine and miconazole treatment on levels of ergosterol, lanosterol, and squalene in Δ*erg5* cells. (A) Levels of ergosterol, lanosterol, and squalene in wild-type cells and Δ*erg5* cells treated with terbinafine or miconazole. Wild-type cells and Δ*erg5* cells were grown to saturation at 27°C in liquid YPD medium. Cells were further incubated at 27°C for 10 h in the absence or presence of terbinafine (0.1, 0.3 µg/ml) or miconazole (0.5, 2.0 µg/ml) as indicated, and then ergosterol, lanosterol, and squalene of the strains were extracted and determined. Error bars represent standard deviations (n = 3 experiments). (B) Effect of terbinafine on the growth of Δ*erg5* cells. Wild-type cells, *hmg1-1*, Δ*spo9*, and Δ*erg5* cells were spotted onto YPD plates or YPD plus terbinafine as indicated and incubated at 27°C for 4 days.

As regards ergosterol, the level in *hmg1-1* mutant was slightly lower than that in wild-type cells as described above (Figure 3 and Figure 6). On the other hand, in Δ*erg6* cells, Δ*erg31*Δ*erg32* cells, and Δ*erg5* cells, the ergosterol levels were extremely lower than that in wild-type cells (Figure 7, Figure 8, and Figure 9A). In addition, both terbinafine and miconazole further decreased the ergosterol levels in all these mutants (Figure 6, Figure 7, Figure 8, and Figure 9A).

As regards lanosterol, the levels in *hmg1-1* mutant, Δ*erg31*Δ*erg32* cells and Δ*erg5* cells were lower than or similar to that in wild-type cells, whereas the level in Δ*erg6* cells was dramatically higher than that in wild-type cells (Figure 6, Figure 7, Figure 8, and Figure 9A). As expected, in all these mutants miconazole increased the lanosterol levels, whereas terbinafine decreased the lanosterol levels (Figure 6, Figure 7, Figure 8 and Figure 9A).

As regards squalene, the level in *hmg1-1* mutant was dramatically lower than that in wild-type cells (Figure 6). However, the squalene levels in Δ*erg6* cells, Δ*erg31*Δ*erg32* cells, and Δ*erg5* cells were almost equal to that in wild-type cells (Figure 7, Figure 8, and Figure 9A). Moreover, terbinafine increased the squalene levels in all these mutants similar to that in wild-type cells. On the other hand, the squalene levels in *hmg1-1* and Δ*erg6* mutants were not affected by miconazole treatment, whereas miconazole increased the squalene levels in Δ*erg31*Δ*erg32* and Δ*erg5* cells (Figure 6, Figure 7, Figure 8 and Figure 9A).

We also examined whether fission yeast cells can grow normally under the condition of lowest ergosterol and lanosterol such as in Δ*erg5* cells with addition of terbinafine. As shown in Figure 9B, the Δ*erg5* cells grew well, similar to wild-type cells, whereas *hmg1-1* mutant and Δ*spo9* cells failed to grow on the YPD plates containing 0.01 µg/ml terbinafine (Figure 9B). These results suggest that some intermediate sterols produced in the isoprenoid pathway may substitute, at least in part for the role of ergosterol in cell growth.

**Figure 10:**
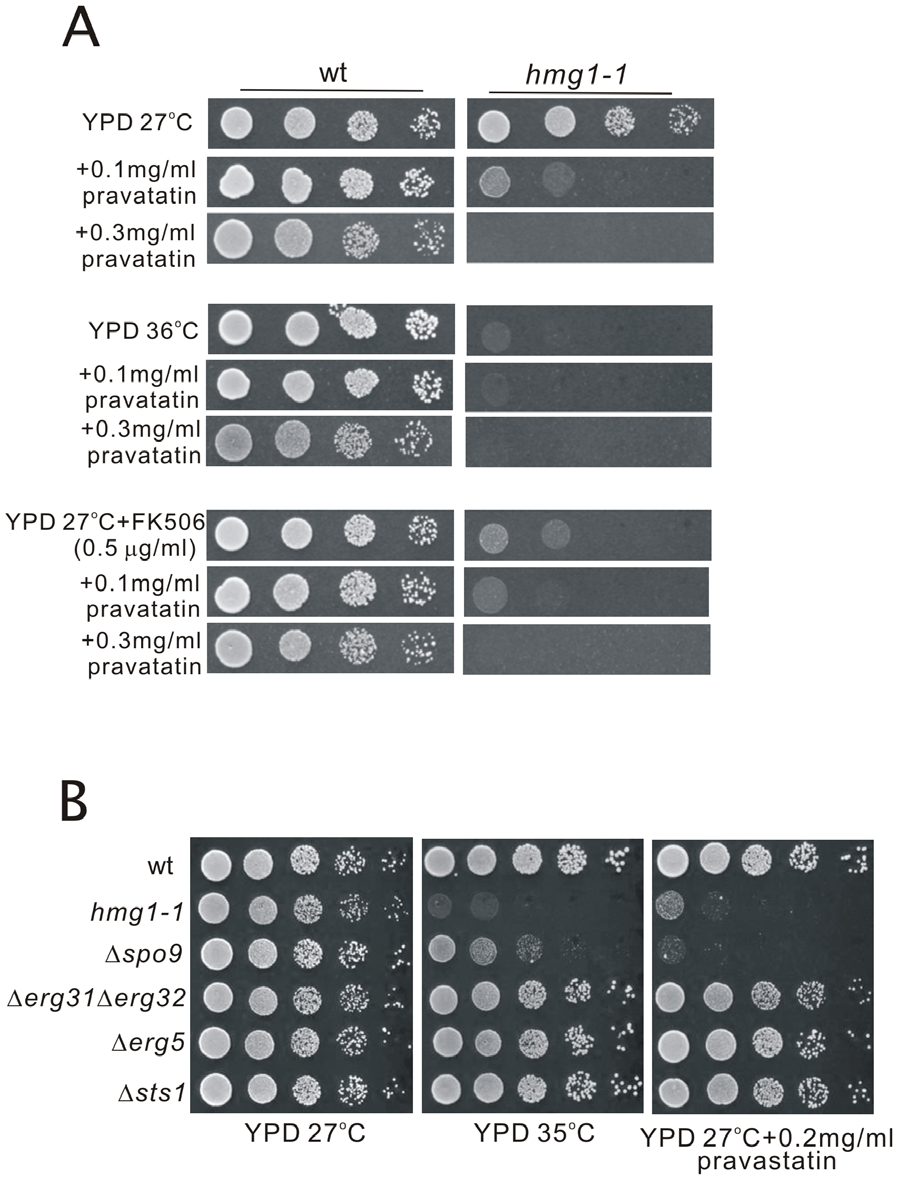
Application of pravastatin to wild-type cells does not cause the similar defects to the phenotypes of *hmg1-1* mutant, and both of the *hmg1-1* mutant and Δ*spo9* cells exhibited overlapping phenotypes. (A) Effect of pravastatin and FK506 on the growth of wild-type cells. Wild-type cells and *hmg1-1* mutants were spotted onto YPD plates or YPD plus pravastatin or/and FK506 as indicated, and then incubated for 4 days at 27°C or at 36°C. (B) Genetic interaction between *hmg1^+^* and *spo9^+^* genes. Wild-type, *hmg1-1*, Δ*spo9*, Δ*erg31*Δ*erg32*, Δ*erg5*, and Δ*sts1* cells were spotted onto each plate as indicated and then incubated for 4 days at 27°C or at 35°C.

As described above, the *hmg1-1* mutant showed hypersensitivities to high temperature and to FK506. Then we tested whether the application of pravastatin to wild-type cells causes the similar defects to these phenotypes of the *hmg1-1* mutant. Results showed that wild-type cells grew well on the YPD plates containing 0.1 mg/ml or 0.3 mg/ml pravastatin at both 27°C and 36°C (Figure 10A). Also, wild-type cells grew well on the YPD plates containing both FK506 and pravastatin at 27°C (Figure 10A), suggesting that the application of pravastatin to wild-type cells does not cause the similar defects to the phenotypes of the *hmg1-1* mutant. As shown in Figure 4B, the levels of FPP and GGPP in wild-type cells were markedly reduced by pravastatin treatment at its high concentration (>2 mg/ml), but these levels were not affected by 0.3 mg/ml of pravastatin treatment (our unpublished data), and growth of wild-type cells was not inhibited at this concentration of pravastatin (Figure 10A). Higher concentrations of pravastatin caused dose-dependent inhibition of their growth, however, no synergistic effect of pravastatin and FK506 was observed (our unpublished data).

In order to investigate the genetic interaction between *hmg1^+^* and the gene encoding an enzyme for the biosynthesis of isoprenoid pyrophosphates such as *spo9^+^*, we compared the phenotypes of *hmg1-1* mutant and Δ*spo9* mutants. Results showed that both of these mutants exhibited overlapping phenotypes, including sensitivities to high temperature and pravastatin (Figure 10B). We also examined whether the other ergosterol-deficient mutants including Δ*erg31*Δ*erg32*, Δ*erg5*, and Δ*sts1* cells exhibited these phenotypes. Results showed that these mutants were not sensitive to high temperature and pravastatin (Figure 10B). These results suggest that the pleiotropic phenotypes in the *hmg1-1* strain might be due to decreased levels of isoprenoid pathway products, possibly isoprenoid pyrophosphates.

## Discussion

Previously, it has been reported that various isoprenoid pathway mutants including *hmg1-1* mutant, Δ*spo9* cells, Δ*erg6* cells, Δ*erg31*Δ*erg32* cells, Δ*erg5* cells, and Δ*sts1* cells exhibited distinct phenotypes such as sensitivities to temperature, FK506, pravastatin, or cycloheximide [6], [9] . In this study, we quantified squalene, lanosterol, ergosterol, FPP, and GGPP in wild-type cells, and the various isoprenoid pathway mutants treated with pravastatin, terbinafine, or miconazole. All of the drugs only slightly decreased ergosterol levels in wild-type cells and *hmg1-1* mutant. However, ergosterol levels in Δ*erg6* cells, Δ*erg31*Δ*erg32* cells, and Δ*erg5* cells were drastically decreased to less than 10% of that in wild-type cells. In wild-type cells treated with pravastatin and *hmg1-1* mutant, quantitative changes of squalene, FPP, and GGPP were larger than that of ergosterol. These results suggest that the pleiotropic effects of statins are caused by quantitative changes of the isoprenoid pathway intermediates such as squalene, lanosterol, FPP, or GGPP rather than ergosterol. We previously reported that the *hmg1-1* mutant exhibited pleiotropic phenotypes, and that ergosterol deficiency is not the primary cause for the phenotypes of *hmg1-1* mutant [9]. In the present study, our results further support this hypothesis, and suggest that the decreased isoprenylation of small GTPases such as Ras, Rho, and Rab may be one of the causes of the pleiotropic effects of statins. However, the mechanisms that cause these effects are still unclear.

Interestingly, squalene is highly produced in Δ*spo9* mutant, but the FPP level is not increased. Spo9 is responsible for catalyzing a reaction from FPP to GGPP [11], and FPP constitutes a branch-point of the isoprenoid pathway. FPP is crucial not only for the biosynthesis of ergosterol but also of other isoprenoid compounds such as dolichols and ubiquinones, and for the feedback regulation of the isoprenoid pathway [12]. Consistent with this notion, our results suggest that FPP serves for the feedback regulation of the isoprenoid pathway, thereby not increased in Δ*spo9* mutant.

Intriguingly, lanosterol levels in Δ*erg6* cells were about 136 folds higher than that in Δ*erg31*Δ*erg32* and 87 folds higher than that in Δ*erg5* cells. Furthermore, the increase of squalene in Δ*erg6* cells treated with terbinafine was higher than that in Δ*erg5* cells, but was similar to that in Δ*erg31*Δ*erg32* cells. Although the reasons for these phenomena are unclear, these results suggest that undefined mechanisms exist in the pathway between squalene and fecosterol catalyzed by Erg6. FPP located at a branch point of the isoprenoid pathway. Squalene and GGPP are synthesized from FPP. Although this branch point is important in the isoprenoid pathway, there may also be important mechanisms related to ergosterol homeostasis in the downstream of squalene because ergosterol is an important cell wall component. Our findings suggest that a cross talk may exist between the downstream of squalene and other pathways. Undefined ergosterol homeostasis mechanisms might be activated by dysfunction in the upstream of the isoprenoid pathway.

In conclusion, the intermediate and final products of the fission yeast isoprenoid pathway were quantified using LC-MS/MS in this study. The findings presented here suggest that the pleiotropic phenotypes caused by the *hmg1-1* mutation and pravastatin might be due to decreased levels of isoprenoid pyrophosphates or other isoprenoid pathway intermediate products, but do not directly arise from a decreased ergosterol level. Our observations provide a further understanding of the regulation of isoprenoid pathway and may also be used as reference value for developing novel drugs that target the isoprenoid pathway.

## Materials and Methods

### Materials

Pravastatin, terbinafine, and miconazole were obtained from Wako Pure Chemical Industries Ltd. (Osaka, Japan), Novartis Pharma K.K. (Tokyo, Japan), Mochida Pharmaceutical Co., Ltd. (Tokyo, Japan), respectively. Squalene, lanosterol, and ergosterol (85.0+% (HPLC)) were purchased from Wako Pure Chemical Industries (Osaka, Japan). Farnesyl pyrophosphate ammonium salt (≥95% (TLC)), and geranylgeranyl pyrophosphate ammonium salt (≥95% (TLC)) were purchased from Sigma-Aldrich, St. Louis, MO, USA. All other reagents were from commercial sources.

### Strains, media, genetic and molecular biology techniques

*Schizosaccharomyces pombe* strains used in this study are listed in Table 1. The complete media, YPD and YES, have been described previously [13], [14]. Standard methods for *S. pombe* genetics were performed as described previously [14]. Gene disruptions are abbreviated by the gene preceded by Δ (for example, Δ*sts1*). Proteins are denoted by roman letters and only the first letter is capitalized (for example, Sts1).

**Table 1:** Strains used in this study.

Strain	Genotype	Reference
HM123	*h^−^ leu1-32*	Our stock
KP207	*h^+^ his2 leu1-32*	Our stock
KP4127	*h^−^ leu1-32 hmg1-1*	[9]
KP4334	*h^−^ leu1-32 ura4-D18 spo9*::*ura4^+^ ade6-M210*	NBRP (FY13124)
KP4248	*h^−^ leu1-32 ura4-D18 sts1*::*ura4* ^+^	[6]
KP4249	*h^−^ leu1-32 ura4-D18 erg5*::*ura4* ^+^	[6]
KP4250	*h^−^ leu1-32 ura4-D18erg31*::*ura4* ^+^ *erg32*::*ura4* ^+^	[6]
KP4251	*h^−^ leu1-32 ura4-D18 erg6*::*ura4* ^+^	[6]

### Extraction of squalene, lanosterol, and ergosterol

Extraction of squalene, lanosterol, and ergosterol from cells was performed essentially as previously described [15], [16] with some modifications. Briefly, cells were grown to saturation in 10 ml of YPD or YES at 27°C, and then one-fifth of the cells were washed two times with ultra pure water, and 1 ml of the extract solvent, chloroform/methanol (2∶1, v/v) including 40 µmol/l pyrogallol and 10 µmol/l pyrene as an internal standard, was added to the mixture. The sample was sonicated for 1 min and mixed overnight. Then, the sample was centrifuged at 16,110 g for 5 min. Subsequently, the supernatant was evaporated for about 30 min using a centrifugal vacuum evaporator. The residue was redissolved in 100 µl of methanol and analyzed by LC-MS/MS.

### Extraction of FPP and GGPP

Extraction of FPP and GGPP from cells was performed essentially as previously described [17], [18] with some modifications. Except for the incubation step, the extraction procedure was performed on ice. Cells were grown to saturation in 10 ml of YPD at 27°C, and then one-third of cells were washed once with 100 mmol/l ammonium hydrogencarbonate solution, then 300 µl of extract solvent (2-propanol/100 mmol/l ammonium hydrogencarbonate solution (1∶1, v/v)) was added to the mixture. The sample was incubated at 70°C for 5 min. The sample was mixed for 1 min, and sonicated for 1 min two times. Furthermore, the same procedure was performed by adding 500 µl of the extract solvent, and then 800 µl of acetonitrile was added to the mixture for deproteinization. The sample was mixed for 10 sec, and was placed on ice for 10 min. Then, the sample was centrifuged at 16,110 g for 5 min. Subsequently, the supernatant was evaporated for about two hours using a centrifugal vacuum evaporator. The residue was redissolved in 100 µl of water/methanol (1∶1, v/v) and analyzed by LC-MS/MS.

### Measurement of squalene, lanosterol, and ergosterol

Squalene, lanosterol, and ergosterol were measured using LC-MS/MS under previously described conditions [19]–[22]. Squalene, lanosterol, ergosterol, and pyrene (as an internal standard) in standard solutions or samples were measured with an alliance HPLC system Waters 2795 Separations Module (Waters, Milford, MA, USA) coupled to an LTQ, linear ion trap with an atmospheric pressure chemical ionization source (Thermo Fisher Scientific, Waltham, MA, USA). An Inertsil ODS-3 column (30 mm×2.1 mm i.d., particle size 2 µm, GL Sciences, Osaka, Japan) was utilized for the separation process. The column temperature was set at 30°C. The mobile phase, consisting of water (solution A) and methanol (solution B), was pumped at a flow rate of 0.25 ml/min. LC separation was performed using a linear gradient program of 70–95% solution B for 2 min, 95–95% solution B for 23 min, 95–70% solution B for 0.1 min, and 70–70% solution B for 4.9 min. A 10 µl aliquot was injected into the column. Analysis was conducted by selected reaction monitoring in the positive ion mode except for pyrene. Pyrene analysis was conducted by selected ion monitoring in the positive ion mode. The heated capillary temperature and the vaporizer temperature were set at 225°C and 450°C, respectively. The discharge current was 5 µA. Ions monitored for each compound are shown in Table 2. The [M+H]^+^ ion was selected as a precursor ion to detect squalene. The [M+H-H_2_O]^+^ ion was selected as a precursor ion to detect ergosterol and lanosterol.

**Table 2:** Ions monitored in the positive ion mode.

No.	Compound	Precursor ion	Product ion	Collision energy
1	Squalene	*m/z* 411.40	*m/z* 231.21	35%
2	Lanosterol	*m/z* 409.38	*m/z* 203.18	35%
3	Ergosterol	*m/z* 379.34	*m/z* 295.24	35%
4	Pyrene	*m/z* 203.09	-	-

### Measurement of FPP and GGPP

FPP and GGPP were measured using LC-MS/MS under previously described conditions [23], [24]. FPP and GGPP concentration in standard solutions or samples were measured with an alliance LC system Waters 2795 Separations Module (Waters, Milford, MA, USA) coupled to an LTQ, linear ion trap with an electrospray ionization source (Thermo Fisher Scientific, Waltham, MA, USA). An XBridge C18 column (50 mm×2.1 mm i.d., particle size 3.5 µm, Waters, Milford, MA, USA) was utilized for the separation process. The column temperature was set at 30°C. The mobile phase, consisting of 20 mmol/L ammonium hydrogencarbonate solution including 0.1% triethylamine (solution A) and Water/Acetonitrile (1∶9, v/v) including 0.1% triethylamine (solution B), was pumped at a flow rate of 0.25 ml/min. LC separation was performed using a linear gradient program of 1–100% solution B for 10 min, 100–100% solution B for 2 min, 100–1% solution B for 0.1 min, and 1–1% solution B for 4.9 min. A 50 µl aliquot was injected into the column. Analysis was conducted by selected reaction monitoring in the negative ion mode. The heated capillary temperature was set at 350°C. The spray voltage was 4.5 kV. Ions monitored for each compound are shown in Table 3. The [M-H]^−^ ion was selected as a precursor ion to detect FPP and GGPP.

**Table 3:** Ions monitored in the negative ion mode.

No.	Compound	Precursor ion	Product ion	Collision energy
1	FPP	*m/z* 381.12	*m/z* 158.93	35%
2	GGPP	*m/z* 449.19	*m/z* 158.93	35%

### Analysis

The peak area ratios of the compounds squalene, lanosterol, and ergosterol to pyrene in mutants or knockout cells were compared to those in wild-type cells. The peak area values of FPP and GGPP in mutants or knockout cells were compared to those in wild-type cells. The level of the respective compounds in wild-type cells was taken as 100%, and the level of the corresponding compounds in mutants or knockout cells was calculated as a percentage of that of the wild-type cells.

### Calibration curve

The calibration curve of each standard was constructed to confirm that LC-MS/MS system used in this study gave correct quantitative data. Each standard solution to construct a calibration curve was prepared as follows. Squalene and lanosterol were dissolved in methanol. The concentrations of the standard solutions of squalene were 1, 5, 10, 50, 100, and 500 µmol/l. The concentrations of the standard solutions of lanosterol were 0.1, 0.2, 0.5, 1, 2, 5, and 10 µmol/l. Pyrene was used as an internal standard. The concentration of the standard solution of pyrene was 90 µmol/l.

FPP and GGPP were dissolved in water/methanol (1∶1, v/v). The concentrations of the standard solutions of both FPP and GGPP were 10, 20, 40, 100, 200, and 400 nmol/l. An internal standard for the quantification of FPP and GGPP was not used.

Ergosterol was quantified absolutely by constructing a calibration curve of Δ*sts1* cell extract (chloroform/methanol (2∶1, v/v)) to serve as blank sample because ergosterol was not detected in Δ*sts1* cells as shown in Figure 1. Standard solutions of ergosterol were prepared by dissolving ergosterol in methanol and by diluting sequentially. The blank sample was prepared by adding methanol to the Δ*sts1* cell extract. The zero reading sample to serve as an internal standard for ergosterol was prepared by adding the pyrene standard solution to the Δ*sts1* cell extract. The samples for constructing the calibration curve were prepared by adding the ergosterol standard solution and the pyrene standard solution to the Δ*sts1* cell extract. The concentrations of ergosterol in the calibration curve samples were 1.9, 3.8, 19, 38, 190, and 380 nmol/mg protein. The concentration of pyrene in the calibration curve samples was 340 nmol/mg protein. The protein concentration of the cell extract was quantified using the Bio-Rad protein assay kit (Bio-Rad Laboratories, Hercules, CA, USA) by Bradford method [25].

## Supporting Information

Figure S1 LC-MS/MS analysis of isoprenoids. A) SRM chromatograms of squalene, lanosterol, ergosterol, and pyrene in the standard solution. B) SRM chromatograms of FPP and GGPP in the standard solution.
(TIF)

Figure S2 Calibration curves of squalene (*r* = 0.9997, A), lanosterol (*r* = 0.9992, B), FPP (*r* = 0.9992, C), and GGPP (*r* = 0.9993, D).
(TIF)

Figure S3 LC-MS/MS analysis of ergosterol in fission yeast extracts. SRM chromatograms of ergosterol (Left) and pyrene (Right) in the blank sample (A), the zero sample (B), and the LLOQ sample (C).
(TIF)

Figure S4 Calibration curve of ergosterol (*r* = 0.9967).
(TIF)
