# Supplementary figures and images for: A Genetic and Pharmacological Analysis of Isoprenoid Pathway by LC-MS/MS in Fission Yeast
Source: PLoS One. 2012 Nov 7;7(11):e49004. doi: 10.1371/journal.pone.0049004 (PMC3492200; doi:10.1371/journal.pone.0049004)

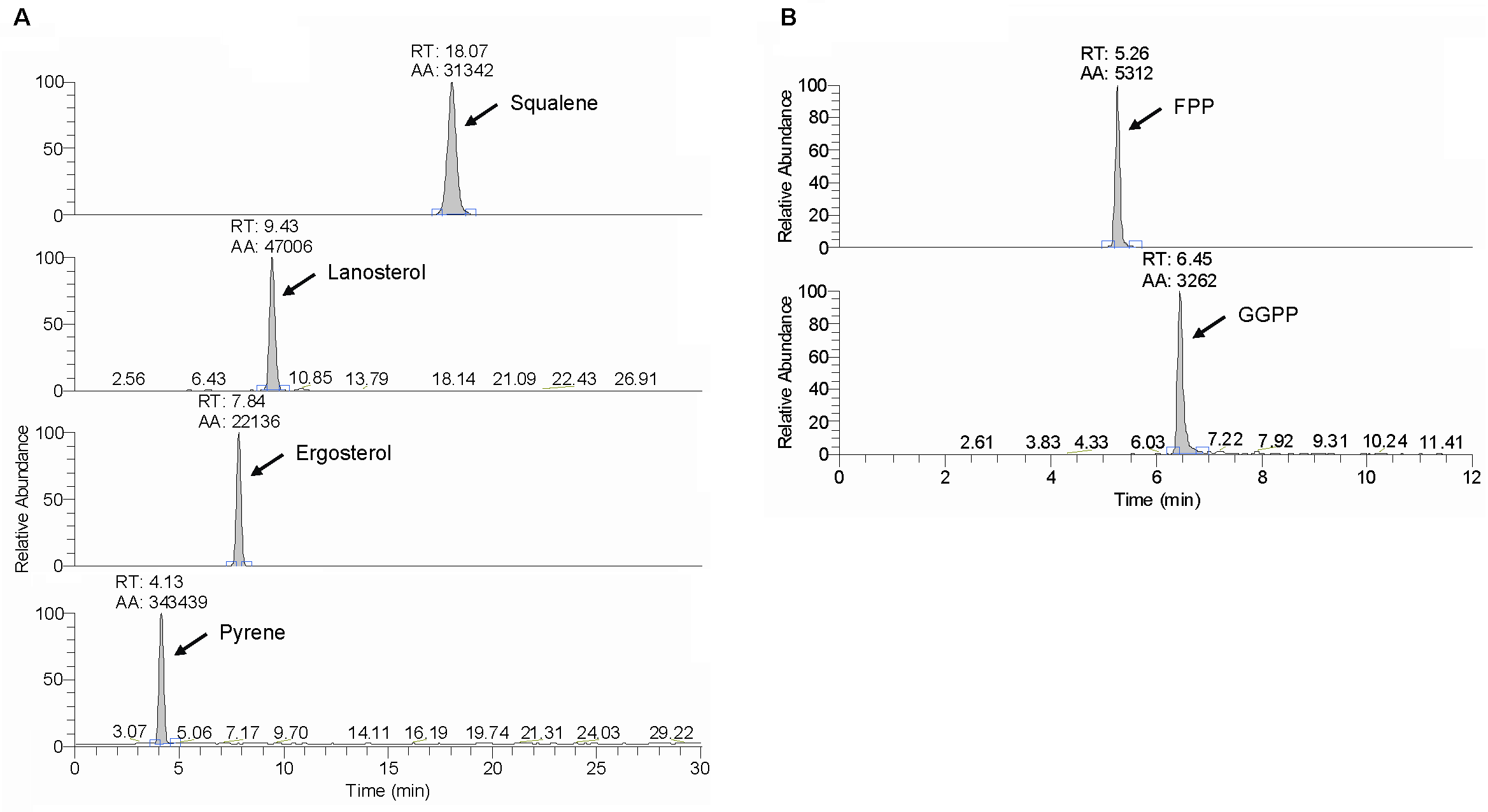

Supplement: Figure S1 — LC-MS/MS analysis of isoprenoids. A) SRM chromatograms of squalene, lanosterol, ergosterol, and pyrene in the standard solution. B) SRM chromatograms of FPP and GGPP in the standard solution. (TIF) [file pone.0049004.s001.tif]

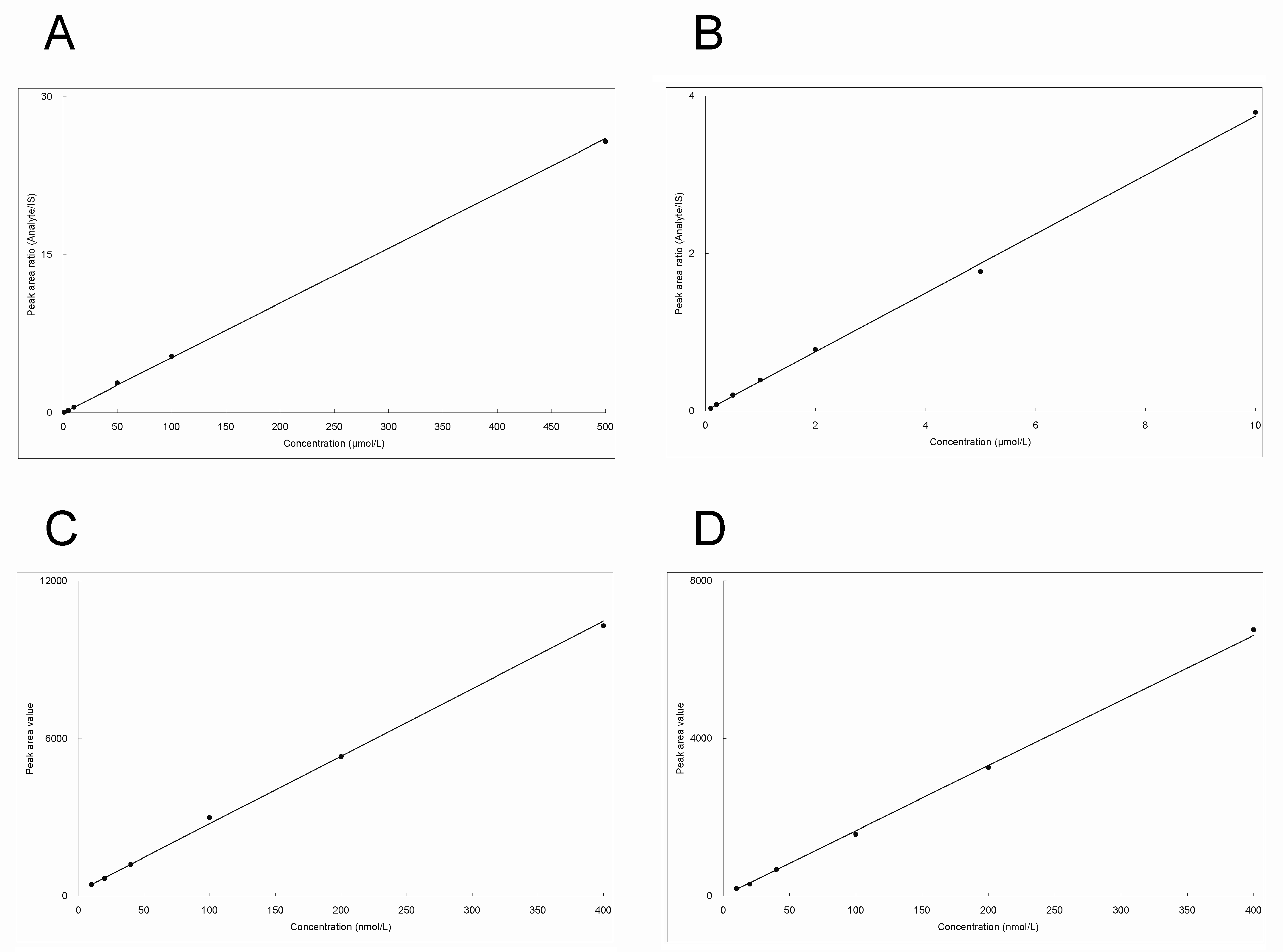

Supplement: Figure S2 — Calibration curves of squalene (r = 0.9997, A), lanosterol (r = 0.9992, B), FPP (r = 0.9992, C), and GGPP (r = 0.9993, D). (TIF) [file pone.0049004.s002.tif]

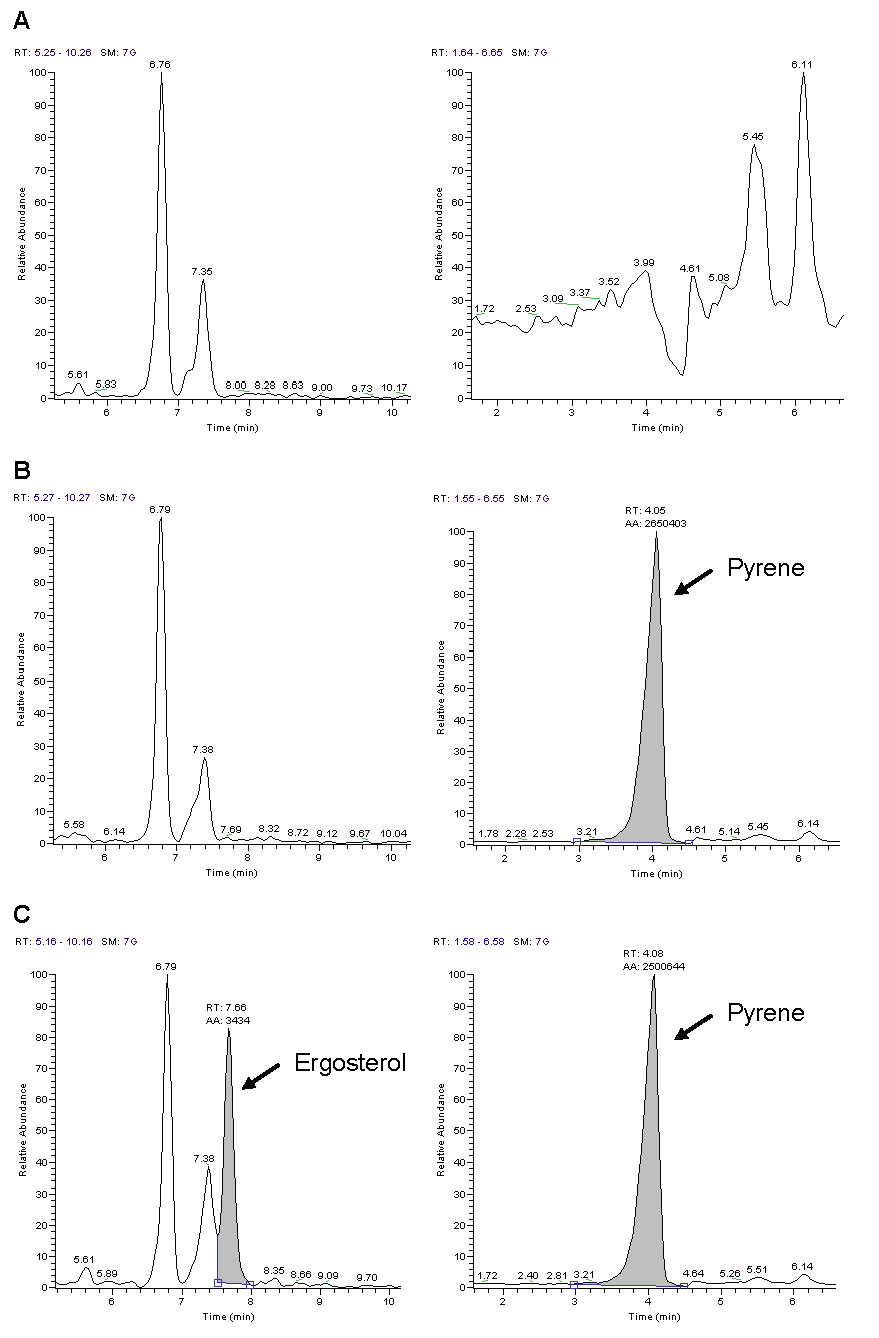

Supplement: Figure S3 — LC-MS/MS analysis of ergosterol in fission yeast extracts. SRM chromatograms of ergosterol (Left) and pyrene (Right) in the blank sample (A), the zero sample (B), and the LLOQ sample (C). (TIF) [file pone.0049004.s003.tif]
